# Economic Benefits from Managing Coffee Berry Borer (*Hypothenemus hampei*) in Hawaii

**DOI:** 10.3390/insects14040350

**Published:** 2023-04-01

**Authors:** Donna Lee, Melissa A. Johnson, Luis F. Aristizábal, Suzanne Shriner, Catherine Chan, Susan Miyasaka, Marisa Wall

**Affiliations:** 1 College of Tropical Agriculture and Human Resources, University of Hawaii at Manoa, Honolulu, HI 96822, USA; djl.donnajlee@gmail.com (D.L.);; 2 Daniel K. Inouye US Pacific Basin Agricultural Research Center, United States Department of Agriculture—Agricultural Research Service, Hilo, HI 96720, USA; 3 Synergistic Hawaii Agriculture Council, Hilo, HI 96720, USA

**Keywords:** bark beetle, *Beauveria bassiana*, integrated pest management, research-based IPM, revenue gain, technology adoption

## Abstract

**Simple Summary:**

Since its introduction to Hawaii in 2010, coffee berry borer (CBB) has dramatically reduced the quality and yield of coffee produced in the islands. We assessed the economic benefits of managing CBB based on three strategies that emerged in Hawaii over the last decade: (1) the use of the entomopathogenic fungus *Beauveria bassiana* alone, (2) early integrated pest management (IPM), which combined monitoring and sanitation with spraying *B. bassiana*, and (3) research-based IPM, which focused on CBB biology in Hawaii, optimization of monitoring, *B. bassiana* applications, and cultural controls. From 2011 to 2021, the economic benefits from managing CBB were USD 52 million using *B. bassiana* alone, USD 69 million from early IPM, and USD 130 million from research-based IPM, for a total of USD 251 million from all management. This suggests that all types of management provide economic benefits to Hawaii growers, but management strategies based on Hawaii-specific research have provided the greatest gains in coffee yield, price, and revenue. Our findings demonstrate that both research and outreach are critical for developing and implementing effective IPM strategies.

**Abstract:**

Coffee berry borer (CBB) is considered the most damaging insect pest of coffee worldwide. CBB was first detected on Hawai‘i Island in 2010, and quickly spread throughout the state’s coffee-growing regions. With the introduction of this pest, Hawaii’s small yet economically important coffee industry was changed forever with growers facing significantly higher production and labor costs, as well as decreased yield and coffee quality. We assessed the economic benefits of managing CBB based on three strategies that emerged in Hawaii over the last decade: (1) the use of the entomopathogenic fungus *Beauveria bassiana* alone, (2) early integrated pest management (IPM), which combined monitoring and sanitation with spraying *B. bassiana*, and (3) research-based IPM, which focused on CBB biology in Hawaii, optimization of monitoring, *B. bassiana* applications, and cultural controls. From 2011 to 2021, the economic benefits from managing CBB were USD 52 million using *B. bassiana* alone, USD 69 million from early IPM, and USD 130 million from research-based IPM, for a total of USD 251 million from all management. Our findings suggest that all types of management provide economic benefits to Hawaii growers, but management strategies based on Hawaii-specific research have provided the greatest gains in coffee yield, price, and revenue.

## 1. Introduction

Coffee is the second most valuable agricultural commodity in Hawaii, with an estimated value of USD 113 million for green coffee and USD 161 million for roasted coffee in 2022 [1]. Although the state’s coffee industry is relatively small compared to other producing regions, the high quality and unique origin of Hawaiian-grown coffee commands premium prices on the world specialty market. For the 2021–2022 growing season, bearing acreage totaled 7100 acres, with 2022 utilized production estimated at 26.7 million pounds (cherry basis) [1]. Most farms in Hawaii are small (<5 acres), family-run operations with annual sales ranging from USD 10,000 to USD 250,000 [2]. While most commercial coffee farms are profitable overall, some lifestyle farms may have a low output-input ratio with negative net profits [3]. Hawaii is unique relative to other coffee-producing regions in that growers have several avenues of distribution and can choose how far to refine their product along the supply chain based on whether they intend to sell to an exporter, a roaster, or directly to consumers.

There are just under 1000 coffee growers in Hawaii [2], and thousands of workers are hired each year to manage and harvest this labor-intensive crop. In 2014, labor accounted for the highest percentage of the production costs for coffee grown in Hawaii at 39%, followed by fertilizers and chemicals (14%) [3]. All coffee grown on Hawai‘i Island is harvested by hand due to the rough terrain and narrow spacing of trees, although there are large plantations on Kaua‘i, O‘ahu, Maui and Moloka‘i that utilize mechanical harvesting. Coffee in Hawaii is cultivated under extremely variable climatic conditions from ~200–800 m elevation on volcanic soils that vary greatly in terms of age and nutrient composition [4]. Almost daily cloud cover in the afternoons provides natural shade during the hottest part of the day, such that most coffee in Hawaii is grown without shade trees, although some farms in Kona have various fruit (avocado, mango, citrus, breadfruit, banana) and nut trees (macadamia) inter-planted with the coffee.

Coffee berry borer (CBB, *Hypothenemus hampei* Ferrari) (Coleoptera: Curculionidae) is widely considered the most damaging insect pest of coffee worldwide [5,6,7]. Hawaii was one of the last coffee-growing regions in the world without an established population of CBB until it was detected in the Kona district of Hawai‘i Island in September 2010 [8]. The tiny beetle rapidly spread across Hawai‘i Island and was detected in the other major growing district of Ka‘ū in May 2011. CBB was later confirmed on the neighboring islands of O‘ahu (2014), Maui (2016), Kaua‘i (2020), and Lāna‘i (2020) [9]. Although it remains unknown exactly how CBB was introduced to the islands, it may have been brought in the clothing or equipment of migrant workers, through improperly fumigated shipments, or by air passengers [10,11,12]. Adult female CBB bore into the coffee fruit (“berry”) and excavate tunnels within the seed (“bean”) to lay their eggs. The developing larvae feed on the endosperm tissue, causing direct damage to the bean. Male and female siblings mate, and the mated females emerge to fly in search of a new berry to infest. The entire life cycle of the CBB is completed within the fruit, making it very difficult to control since the female is only vulnerable to insecticide sprays when it leaves the natal fruit.

The arrival of this global pest completely changed Hawaii’s coffee industry forever. Infestation in poorly managed farms reached as high as 95% [13]. The yield and quality of Hawaiian coffee decreased, and significant declines in “Fancy” and “Extra fancy” grades of coffee beans were seen in the years that followed [14,15]. Large processors implemented quality assessments for each bag of cherry delivered, with the grower receiving 10–15 cent reductions for infestation above 10%. Production and labor costs increased due to the need to conduct strict sanitation practices and to apply insecticides to minimize losses [14,15]. For the 2011/12 and 2012/13 seasons, the impact from CBB was estimated at USD 25.7 M in lost sales, USD 12.7 M in crop loss, USD 7.6 M in lost household earnings, and a loss of more than 380 jobs [16].

When CBB was first reported in Hawaii, information from other coffee producing countries was identified for incorporation into an integrated pest management (IPM) program. The key components of this program include monitoring, cultural control practices, and the use of biological control agents [17,18,19,20]. The first major milestone in the development of a Hawaii-specific IPM came in 2011, when the GHA strain of *Beauveria bassiana* (Basl. Criv.) Vuill. (Hypocreales: Cordycipitaceae) was approved for use on Hawaiian coffee (sold commercially as BotaniGard^®^ ES and Mycotrol^®^ ESO; Lam International Corporation, Butte, MT, USA). This entomopathogenic fungus is one of most important natural enemies of CBB and can result in mortality levels of up to 70% [6,14]. 

A second important milestone was introduction of the 30-tree sampling method [17] in 2012 for CBB monitoring. This allowed growers to estimate infestation percentage, determine CBB position within the berry, and locate hotspots of CBB activity, all of which are used to inform spray decisions [14,15,21]. The following year (2013) several organizations (University of Hawaii at Manoa (UHM) College of Tropical Agriculture and Human Resources (CTAHR), Synergistic Hawaii Agricultural Center (SHAC), Hawaii Department of Agriculture (HDOA), United States Department of Agriculture–Agricultural Research Service (USDA–ARS), and numerous coffee grower associations) came together to participate in a CBB summit, with the purpose of developing IPM recommendations for controlling CBB in Hawaii. The resulting document provided growers with information on best practices for monitoring, pruning, spraying *B. bassiana*, harvesting, and strip-picking [22]. We term this combination of practices the “early IPM” for CBB in Hawaii for the purposes of this study. A second CBB summit in 2014 further solidified early IPM recommendations and updated guidelines [23]. 

The last major milestone in the success of the early IPM was establishment of a *B. bassiana* subsidy program funded by HDOA, SHAC, and USDA-FAS. This program reimbursed growers for up to 75% of the costs associated with spraying approved *B. bassiana* products (up to USD 9000 per farmer/year) from 2014 to 2016. The program was extended several times, with the most recent programs (2018–2026) reimbursing growers for up to 50% of costs or up to USD 6000 per farmer/year. Since the inception of the subsidy program, the use of commercial *B. bassiana* products on Hawaii coffee farms increased from 80% of farms in 2013, to 85% of farms in 2015, and 95% of farms in 2016. Among surveyed farmers, ~95% found *B. bassiana* to be effective. Approximately 30% of surveyed farmers responded that without the subsidy, they would stop growing coffee or strongly consider terminating it as a crop [24]. 

Although the early IPM provided a starting point for CBB management in Hawaii, there were many unknowns regarding the basic biology of CBB under Hawaii’s unique environmental and agroecological conditions. In addition, the high production and labor costs and severe shortage of labor created major challenges for managing this new pest in a way that was economically feasible for growers. Several federal and state agencies, as well as farmer associations and coffee industry professionals worked together to address these issues and improve upon the early IPM based on scientific data collected in the islands. These studies resulted in a better understanding of CBB infestation patterns and flight activity [13,25,26], development times [27], post-harvest CBB reservoirs [28], efficacy of cultural control practices [15,29,30], simplification of monitoring strategies [21,31,32], identification of potential biocontrols [33,34], and efficacy and optimization of *B. bassiana* applications [35,36,37,38,39]. New information from these studies was incorporated into updated IPM guidelines beginning in 2016 [40,41], which we refer to as “research-based IPM” for CBB in Hawaii.

In the present study, our aim was to estimate the economic benefits of managing CBB in Hawaii under three scenarios: (1) the use of *B. bassiana* alone; (2) the implementation of early IPM, which combined monitoring and sanitation with spraying *B. bassiana*; and (3) the use of research-based IPM, which provided insights into CBB development, flight behavior, infestation patterns, monitoring techniques, and spray optimization. Findings from this study support the continued development and adoption of IPM strategies, which will increase yields, quality, and revenue to improve the livelihoods of coffee growers in Hawaii.

## 2. Methods

To estimate economic benefits under the three scenarios, we first estimated the statewide and regional coffee-bearing acreage from 2006 to 2021 and then used CBB detection dates for each growing region to estimate the infested acreage over time. The rate at which CBB management recommendations were adopted by farmers in Hawaii was then estimated using Rogers’ theory of technology adoption [42]. We then modeled the impact of CBB infestation and management on coffee production and coffee prices. Lastly, to quantify the economic benefits from CBB research and management, we estimated and compared average gains in statewide coffee yield, price, and revenue with and without CBB management. 

### 2.1. Hawaii Coffee Acreage (2006–2021) and Infestation (2010–2021) 

#### 2.1.1. Statewide and Regional Coffee Acreage

**Figure 1:**
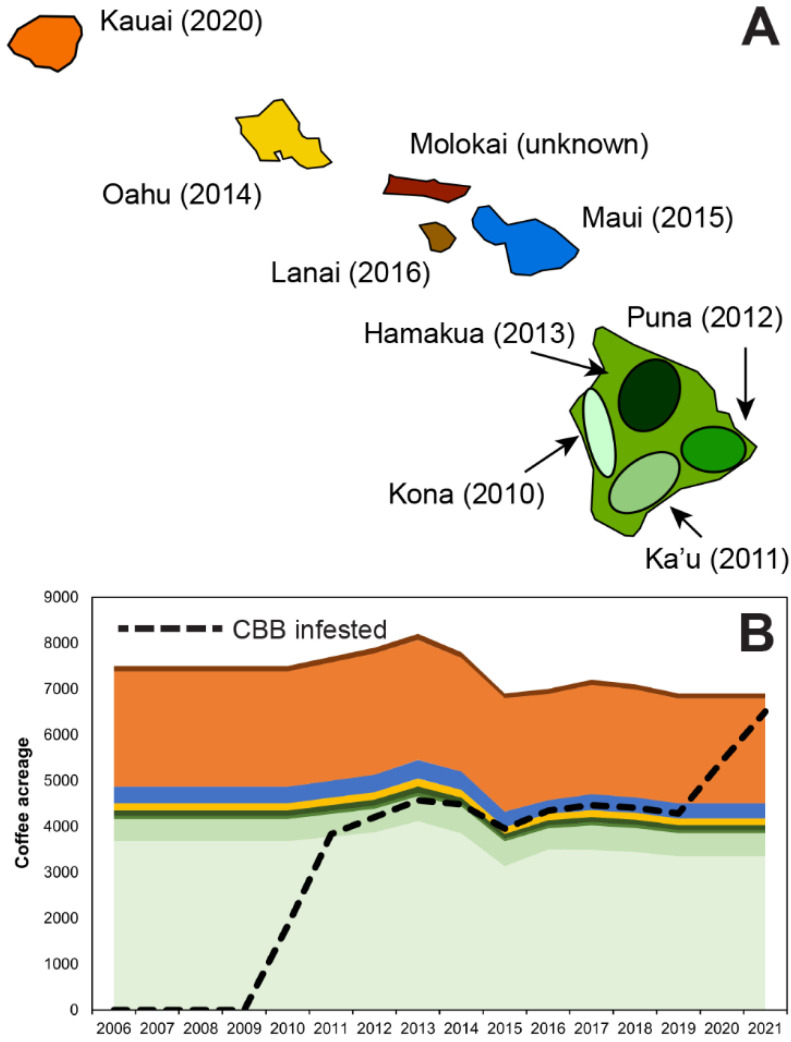
(**A**) Map of Hawaiian Islands showing initial coffee berry borer (CBB) infestation dates (τ_h_) in each coffee-growing region (*h*), and (**B**) Hawaii statewide and regional coffee acreage from 2006 to 2021, and CBB infested acreage following its introduction in 2010. Colors correspond to regions shown on the map. Note that Lanai primarily has wild coffee and no significant commercial coffee farms, and is thus not shown in B.

Statewide bearing coffee acreage in Hawaii has been reported annually by the USDA National Agriculture Statistics Service since 1946. However, the State of Hawaii does not routinely report regional coffee acreage and has not previously attempted to measure CBB infested acreage. To model CBB spread, we developed a procedure to estimate both regional acreage and statewide infested acreage over time. From 2013 to 2016, the state reported regional bearing coffee acreage for most of the nine regions, but not every region and not every year. For our 16-year time-period (2006–2021) and nine growing regions (Kona, Ka‘ū, Puna, Hāmākua, Oah‘u, Maui, Lāna‘i, Kaua‘i, Moloka‘i; see Figure 1A), there were 19 acreage observations and hence 109 missing data points (Table S1). 

Statewide coffee acreage was relatively steady during this time-period and not trending. This allowed us to confidently estimate the missing data points by extrapolating backwards to 2010, forwards to 2021, and interpolating where missing values were between years when acreage was reported. In this way, we generated a first approximation of regional bearing coffee acreage, a’_ht_, for each region *h* = Kona, Ka‘ū, Puna, Hāmākua, Oah‘u, Mau‘i, Lana‘i, Kaua‘i, and Moloka‘i, for years *t* = 2010 to 2021(see Table S2; Figure 1B).

We then summed a’_ht_ over all regions to obtain A’_t_:

A’_t_ = Σ_h_ a’_ht_ (1)

We compared the value of A’_t_ to official statewide acres, A_t_, and as expected, A’_t_ ≠ A_t_. To make the correction, we generated a calibration factor *φ_t_* for each year *t*:

*φ_t_* = A_t_/A’_t_ (2)

We multiplied the calibration factor *φ_t_* by a’_t_ to obtain estimated regional acreage a_ht_:

a_ht_ = *φ_t_* a’_ht_ (3)

Summing a_ht_ over *h* yielded A”_t_:

A”_t_ = Σ_h_ a_ht_ (4)

We compared A”_t_ and A_t_ and verified that:

A”_t_ = A_t_ (5)

Meaning that our estimated values of regional coffee acreage a_ht_ sum to reported statewide coffee acreage A_t_:

A_t_ = Σ_h_ a_ht_ (6)

Estimates of regional coffee acreage over time are shown in Figure 1B and Table S2.

#### 2.1.2. Regional CBB Infested Acreage—CBB Spread over Time

With estimated regional coffee acreage and the dates when CBB was detected in each region, we can begin to estimate infested acreage over time. First, we define τ_h_ to be the year CBB was detected in region *h*, as shown in Figure 1A. During the first year of detection, we assume 50% of the acreage is infested. During the second year we assume 95% of the acreage is infested. This approximation was provided by our expert panel comprised of Hawaii coffee growers (T. Greenwell, S. Shriner, and M. Bondera), coffee processors (T. Greenwell and S. Shriner), extension experts (L. Aristizábal, M. Bondera, and S. Shriner), and CBB researchers (M. Johnson and L. Aristizábal). Estimates mimic a fast exponential rate of spread, which is consistent with expert panel observations. The proportion of infested acreage in region *h* at time *t* is given by α_ht_ = 50% for *t* = τ_h_ and α_ht_ = 95% for *t* > τ_h_+1. Using the values for α_hτ_ and regional acreage a_ht_ we can compute CBB infested acreage i_ht_:

i_ht_ = α_ht_ a_ht_ (7)

We sum i_ht_ over all *h* to obtain statewide infested acres I_t_:

I_t_ = Σ_h_ i_ht_ = Σ_h_ α_ht_ a_ht_ (8)

Our estimate of regional infested acres appears in Figure 1B and Table S3. 

### 2.2. Technology Adoption

#### 2.2.1. Applying Rogers’ Technology Adoption Theory to CBB Management

To estimate the rate at which CBB management recommendations were adopted by farmers in Hawaii, we applied Rogers’ theory of technology adoption [42] which divides the population of any group into innovators, early adopters, early majority, late majority, and laggards, where the innovators (0.025) and early adopters (0.135) are the first to undertake any new technology, followed by early majority and late majority. The laggards (0.16) may never adopt the new technology. Here, we define μ_ht_ to be the proportion of CBB infested acres in region *h* that is managed. As defined previously, τ_h_ is the year CBB was first detected in region *h*. For *t* < τ_h_ management is not needed so μ_ht_ = 0. For *t* = τ_h_ innovators and early adopters begin managing for CBB, therefore μ_ht_ = 0.16. Each year, more farmers adopt CBB management practices up through year three when 84% of the infested acreage is managed. Parameter values for μ_ht_ are as follows: for *t* < τ_h_, μ_ht_ is zero; at *t* = τ_h_, μ_ht_ = 0.16; at *t* = τ_h_ + 1, μ_ht_ = 0.23; at *t* = τ_h_ + 2, μ_ht_ = 0.50; at *t* = τ_h_ + 3, μ_ht_ = 0.84; and at *t* > τ_h_ + 3, μ_ht_ = 0.84. With μ_ht_ we can compute regional managed infested acreage m_ht_ as follows:

m_ht_ = μ_ht_ i_ht_ (9)

Summing m_ht_ over *h* gives us statewide managed infested acres M_t_:

M_t_ = Σ_h_ m_ht_ (10)

#### 2.2.2. Managed and Unmanaged Infested Acres over Time—By Management Type

To capture the evolving management technology, we define *j* to be the type of management applied, where *j* = B, C, D, E, F. Here, B refers to the use of *B. bassiana* alone to control CBB, C represents early IPM management following recommendations that came out in 2013–2015, D represents management that follows research-based IPM recommendations that came out in 2016–2021, E represents no management of infested acreage, and F represents no management of uninfested acreage.

**Figure 2:**
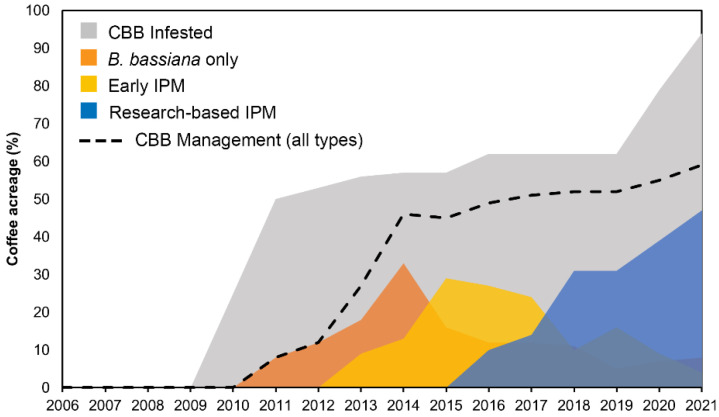
Coffee berry borer (CBB) infested acreage as a proportion of all coffee acreage in Hawaii (gray), acreage for each of the three management types (*B. bassiana* only, Early IPM, and Research-based IPM) as a proportion of infested acreage, and total CBB managed acreage as a combination of all three management types (dashed line).

Recall that total statewide infested acreage is I_t_ and total managed infested acres is M_t_. We introduce a new term υ_jt_ to be the proportion of infested acres I_t_ managed by each type *j* = B, C, D. For the first three years, 2010–2012, we assume all managed acreage are using *B. bassiana* only (type B). During 2013–2015, we apply Rogers’ theory of technology adoption assuming the adoption rate for early IPM (type C) to be 0.16 in the first year, 0.23 in the second year, and 0.50 in the third year. During 2016–2018, we apply Rogers’ theory of technology adoption assuming the adoption rate for research-based IPM (type D) to be 0.16 in the first year, 0.23 in the second year, 0.50 in the third year and so on. For years 2016–2019, we computed values of *B. bassiana*-only usage from survey data collected as part of the HDOA *B. bassiana* subsidy program (M. Bondera, pers. comm.) as 0.19, 0.20, 0.18, and 0.086. The remaining values for υ_jt_ for *j* = B, C, D were computed as 1 = υ_Bt_ +υ_Ct_ + υ_Dt_ (see Table S4). The numerical results for acreage by management type *j* as a proportion υ_jt_ of infested acres I_t_ are shown in Figure 2 and Table S5.

#### 2.2.3. All Coffee Acreage—Infested and Uninfested, Managed and Unmanaged

Coffee acreage A_t_ is either infested I_t_ or uninfested U_t_:

A_t_ = I_t_ + U_t_ (11)

Infested acres I_t_ are either managed M_t_ or unmanaged N_t_:

I_t_ = M_t_ + N_t_ (12)

where υ_Bt_, υ_Ct_, and υ_Dt_ are the proportion of infested acres under each management type then I_t_ can be written as:

I_t_ = (υ_Bt_, υ_Ct_, and υ_Dt_) I_t_ + N_t_ (13)

A_t_ can be written as:

A_t_ = (υ_Bt_, υ_Ct_, and υ_Dt_) I_t_ + N_t_ + U_t_ (14)

In addition, A_t_ can be rewritten as:

A_t_ = (υ_jt_ I_t_ + υ_jt_ I_t_+ υ_jt_ I_t_) + N_t_ + U_t_ (15)

Dividing both sides of the previous equation by A_t_ gives us:

1 = [(υ_jt_ I_t_ + υ_jt_ I_t_+ υ_jt_ I_t_) + N_t_ + U_t_]/A_t_ (16)

or

1 = υ_jt_ I_t_/A_t_ + υ_jt_ I_t_/A_t_ + υ_jt_ I_t_/A_t_ + N_t_/A_t_ + U_t_/A_t_ (17)

which we can write as:

1 = ω_Bt_ + ω_Ct_ +ω_Dt_ +ω_Et_ +ω_Ft_ (18)

where ω_jt_ is the proportion of acreage A_t_ for *j* = B, C, D, E, F. Computation of values for ω_jt_ are displayed in Table S6.

### 2.3. Economic Approach: Impact of Infestation and Management on Coffee Yield and Price

#### 2.3.1. Production and Economics

Here we model the impact of CBB infestation on coffee production and coffee prices. We assume that higher infestation levels cause a reduction in yields and price. To ascertain the relationships between infestation, yields, and coffee prices, we again consulted our expert panel (see above). We further assume that management reduces infestation and helps to improve yields and prices, and the more effective the management, the lower the infestation level.

#### 2.3.2. Economic Model Scenarios

The economic model to evaluate the impact of infestation levels on yield and price are basic production and price functions between acreage, yield, prices, and management type. The baseline model replicates observed production and available management types. To generate the baseline, we used estimated regional acreage, estimated infested acreage, and rate of adoption for the three management types. Yields and prices were based on infestation and management type. Total production was based on acreage and yields. Total revenue was based on production and prices. 

#### 2.3.3. Coffee Acreage A_t_

Estimates of coffee acreage *a*_jt_ for each management type *j* and time *t* were calculated using:

*a*_jt_ = ω_jt_ A_t_ (19)

Using the equation above, the values in Table S5 for A_t_ and values in Table S6 for ω_jt_ we obtain the distribution of acreage for uninfested, infested, managed, and unmanaged acres (Table S5).

#### 2.3.4. Coffee Yield Y_t_

Official reported statewide yields are based on total production and estimated acreage. However, across the landscape actual farm yields vary widely depending on several factors. For this analysis, we focus on yield variation as a function of CBB infestation. On average, coffee yields diminish with rising CBB infestation and increase with improved CBB management. More effective management practices help to preserve yields. While we cannot directly observe CBB infestation levels statewide, we relied on opinion from our expert panel to calculate implied relative yields. We define y_jt_ to be the average yield per acre under each management type *j* at time *t* and define ψ_j_ to be a parameter that captures relative yield.

y_jt_ = j^i^_j_ y_Ft_ (20)

Here y_Ft_ is the coffee yield from uninfested acreage and y_jt_ is yield for all other *j*. From our expert panel, values for ψ_j_ are as follows: infested (ψ_F_) =1, infested unmanaged (ψ_E_) = 0.5, managed by *B. bassiana* only (ψ_B_) = 0.75, managed by early IPM (ψ_C_) = 0.85, and managed by research-based IPM (ψ_D_) = 0.95. Recall that ω_jt_ represents the proportion of total coffee acreage that is infested, uninfested, managed, and unmanaged. We can derive the value of y_Ft_ as follows:

We write coffee yield, Y_t_, as the proportion of acreage, ω_jt_, multiplied by average yield, y_jt_, for j = B, C, D, E, F:

Y_t_ = ω_Bt_ y_Bt_ + ω_Ct_ y_Ct_ + ω_Dt_ y_Dt_ + ω_Et_ y_Et_ + ω_Ft_ y_Ft_ (21)

Into Equation (21), we substitute y_jt_ = ψ_j_ y_Ft_ for all y_jt_ (see Equation (19)):

Y_t_ = ω_Bt_ψ_B_ y_Ft_ + ω_Ct_ψ_C_ y_Ft_ + ω_Dt_ ψ_D_ y_Ft_ + ω_Et_ ψ_E_ y_Ft_ + ω_Ft_ψ_F_ y_Ft_ (22)

We rearrange Equation (22) to solve for coffee yield from uninfested acreage, y_Ft_:

y_Ft_ = Y_t_/(ψ_B_ ω_Bt_ + ψ_C_ ω_Ct_ + ψ_D_ ω_Dt_ + ψ_E_ ω_Et_ + ψ_F_ ω_Ft_) (23)

We can compute y_Ft_ using ω_Bt_ in Table S6, ψ_j_ as described in the previous paragraph, and Y_t_ in Table 1. Then we calculate all other average yield per acre under each management type *j* at time *t*, y_jt_, using:

y_jt_ = ψ_j_ y_Ft_ (24)

This method for calibrating yields preserves reported statewide yields since the weighted average of the calibrated yields equals statewide reported yields. Computed baseline yield values y_jt_ are displayed in Table 1.

#### 2.3.5. Coffee Price P_t_

Average coffee prices are reported annually by the USDA-NASS based on total revenue and total production. Actual prices received by farmers for their crops vary widely depending on coffee quality, supply, and demand. For this analysis, we assumed price decreases with increasing level of CBB infestation. While we do not observe CBB infestation of the coffee sold, we know CBB management reduces infestation [13,15], which in turn, results in higher prices. Here we define p_jt_ to be price per pound of parchment received under management status *j* and ρ_jt_ to be the relative price parameter such that:

p_jt_ = ρ_j_ p_Ft_ (25)

Here p_Ft_ is the premium price received per pound for uninfested parchment. Values of ρ_jt_ are ρ_Bt_ =87%, ρ_Ct_ =93%, ρ_Dt_ =100%, ρ_Et_ = 45%, and ρ_Ft_ =100%. We know that revenue is:

P_t_ Q_t_ = Σ_j_ p_jt_ q_jt_ = p_Bt_ q_Bt_ + p_Ct_ q_Ct_ + p_Dt_ q_Dt_ + p_Et_ q_Et_ + p_Ft_ q_Ft_ (26)

Here P_t_ is reported price, Q_t_ is reported production, values for q_jt_ are computed (q_jt_ = y_jt_*a*_jt_) using values of average yield y_jt_ (Table 1) and estimated regional acreage *a*_jt_ (Table S5).

Into the equation above, we substitute Equation (24), and obtain the expression:

P_t_ Q_t_ = ρ_B_ p_Ft_ q_Bt_ + ρ_C_ p_Ft_ q_Ct_ + ρ_D_ p_Ft_ q_Dt_ + ρ_E_ p_Ft_ q_Et_ + ρ_F_ p_Ft_ q_Ft_ (27)

We can rewrite the above equation to solve for p_Ft_:

p_Ft_ = P_t_ Q_t_/(ρ_B_ q_Bt_ + ρ_C_ q_Ct_ + ρ_D_ q_Dt_ + ρ_E_ q_Et_ + ρ_F_ q_Ft_) (28)

We compute premium price per pound for uninfested parchment, p_Ft_, and the corresponding values of p_jt_ = ρ_jt_ p_Ft_ for the remaining *j*. This calibration method preserves reported statewide prices since the weighted average of the calibrated price equals the statewide reported price. Computed baseline price values are displayed in Table 2.

### 2.4. Hypothetical Scenario for Benefits Estimation

To quantify the value of CBB research, extension, and management, we developed a hypothetical scenario following the arrival of CBB in Hawaii in 2010. In the hypothetical scenario, there are no statewide efforts or responses from the government, coffee associations, or the university to help farmers manage the crop pest. Outreach, recommendations, subsidies, research, and strategy development are all absent. 

#### 2.4.1. Scenario Assumptions

We assumed that CBB arrived in Hawaii and spread across the islands at the same rate as in the baseline (observed). This is a simplifying and conservative assumption as some management recommendations include measures designed to slow the rate of spread within a farm, across farms, and across regions. In the absence of management, it is possible that CBB might have spread faster. However, we feel this is a reasonable assumption given the relatively small (16%) rate of technology adoption in the first year of infestation and fast observed rate of CBB spread even with management. We assume yields for uninfested (y_F_) and infested (y_E_) coffee are the same as the values computed for the baseline, that is infested coffee yield is 50% of uninfested yield (i.e., ψ_E_ = 0.50 and y_E_ = ψ_E_ y_F_)_._ We feel this is a reasonable assumption based on expert panel observations. We assume price for uninfested (p_F_) and infested (p_E_) coffee are the same as the values computed for the baseline, that is price for infested coffee is 45% of uninfested coffee i.e., ρ_E_ = 0.45 and ρ_E_ = ρ_E_ρ_F_. This is a simplifying assumption and under this scenario, the price for infested and uninfested coffee could be higher or lower. Roasters may be willing to pay more for both low- and high-quality coffee if the high-quality coffee is in short supply. In addition, the price for low-quality coffee might be relatively closer to the price of high-quality coffee in which case for any given year in the past, the benefit from management could be slightly less than estimated. We assume bearing acreage A_t_ remains the same as in the baseline. Uninfested acreage U_t_ and infested acreage I_t_ are as shown in Table S5. Acreage continues to be in flux, but we assume the acreage that goes in and out of production each year is as observed (Table S5). Our estimate is conservative in that our model does not simulate an increase in land going out of production due to unmanaged infestation, reduced yields, and reduced quality. Instead, we assume revenues cover long-term costs, and total acreage remains the same.

#### 2.4.2. Coffee Yield

In our hypothetical “no management” scenario beginning in 2010, yields on uninfested acreage averaged 1155 lbs per acre ranging from a high of 1337 lbs per acre in 2010 to a low of 897 in 2016. Hypothetical yields on infested acreage averaged 578 lbs per acre, with a high of 669 lbs per acre in 2010 and a low of 449 lbs per acre in 2016. Annual coffee yields in the hypothetical scenario are displayed in Table 1.

#### 2.4.3. Coffee Price

In our hypothetical “no management” scenario beginning in 2010, the price for uninfested coffee ranges from a high of USD 14.78 per lb in 2019 to a low of USD 3.60 per pound in 2009. For infested coffee, the price ranges from a high of USD 6.65 per lb in 2019 to a low of USD 1.85 per lb in 2010. Annual average prices in the hypothetical scenario are displayed in Table S7.

#### 2.4.4. Benefits Estimation

To quantify the benefits from CBB management, we estimated and compared average gains in statewide coffee yield, price, and revenue with and without each of the three types of CBB management. Average gain in yield per acre across all coffee farms was computed as follows:

Yield Gain (t) = Y_t_ − (y_Ft_ U_t_ + y_Et_ I_t_)/A_t_ (29)

% Yield Gain (t) = Yield gain (t)/Y_t_ (30)

Average gain in price per pound across all coffee farms was computed as follows:

Price Gain (t) = P_t_ − (p_Ft_ U_t_ + p_Et_ I_t_)/A_t_ (31)

% Price Gain (t) = Price Gain (t)/P_t_ (32)

Average gain in revenue per acre across all coffee farms was computed as follows:

Average Revenue Gain (t) = P_t_Y_t_ − (p_Ft_ y_Ft_ U_t_ + p_Et_ y_Et_ I_t_)/A_t_ (33)

% Average Revenue Gain (t) = Revenue Gain (t)/P_t_Y_t_ (34)

Annual statewide revenue gain across all coffee farms was computed as follows:

Total Revenue Gain (t) = P_t_ Y_t_ A_t_ − (p_Ft_ y_Ft_ U_t_ + p_Et_ y_Et_ I_t_) (35)

% Total Revenue Gain (t) = Total Revenue Gain (t)/P_t_ Y_t_ A_t_ (36)

## 3. Results

From 2006 to 2021, statewide bearing coffee acreage in Hawaii ranged from a low of 6300 acres in 2006 to a high of 8200 acres in 2013 (Table S5; Figure 1B). From 2010 to 2011, CBB spread rapidly throughout Hawaii Island’s two main growing regions of Kona and Ka’u, which together account for ~50% of the coffee acreage in the state (Table S3; Figure 1B). A second large increase in infested acreage was estimated in 2020 (Table S3; Table S5) following the detection of CBB on Kauai, which has more than 3000 acres of commercially grown coffee (Figure 1A,B).

During the first two years of the invasion, 8–12% of the infested acreage was managed using *B. bassiana* alone while the remaining ~40% of infested acreage went unmanaged (Figure 2). In 2013, 18% of infested coffee was managed using *B. bassiana* alone and 9% was managed using the newly introduced early IPM (Figure 2). Management of CBB using only *B. bassiana* peaked at 33% in 2014, while the use of early IPM strategies peaked at 29% in 2015. Research-based IPM began to be implemented by growers in 2016, with the use of these science-backed strategies increasing steadily over the next five years. By 2021, 47% of infested acreage was managed using research-based IPM compared to only 8% using *B. bassiana* alone and 4% using early IPM (Figure 2).

**Figure 3:**
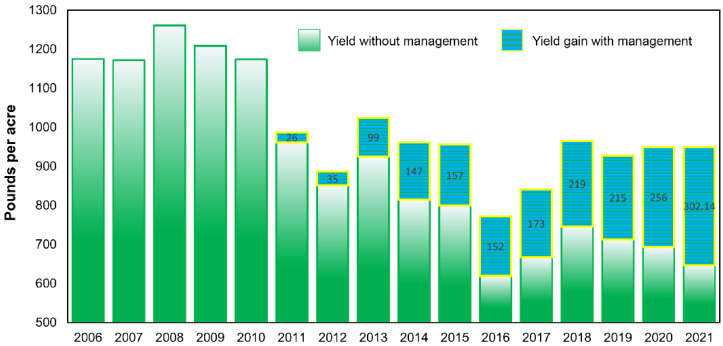
Average coffee yield (pounds of parchment coffee per acre) across all Hawaii farms from 2006 to 2021 and gain in yield due to all combined CBB management beginning in 2011.

From 2006 to 2021, coffee yields ranged from a high of 1261 pounds of parchment per acre in 2008 to a low of 771 pounds per acre in 2016 (Figure 3; Table 1). Average yield during the five-year period prior to CBB introduction was 1198 lbs/acre, compared to an average of 963 lbs/acre the first five years after CBB was introduced (20% reduction). Average yield gains due to CBB management increased from 26 lbs/acre (2.6% increase) in 2011 to 302 lbs/acre (31.8% increase) in 2021 (Figure 3; Table S8). 

**Figure 4:**
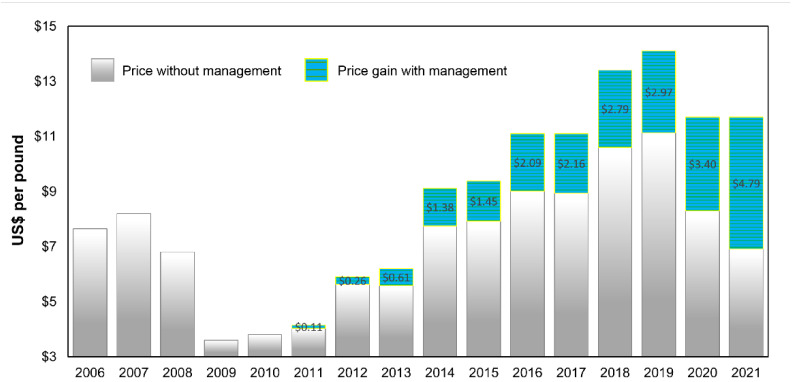
Average price per pound (USD) of parchment coffee without CBB management in Hawaii from 2006 to 2021 and gain in price due to all combined CBB management beginning in 2011.

Over the 16-year sampling period, coffee prices ranged from a low of USD 3.60 per pound in 2009 to a high of USD 14.10 per pound of parchment in 2019 (Figure 4; Table S9). Prior to the CBB invasion, the observed average price per pound for parchment coffee was USD 6.01, compared to an average of USD 6.95 per pound in the five years post-invasion (16% increase). Our results showed average price gains from all CBB management combined rising from USD 0.11 per pound (2.7% increase) in 2011 to USD 4.79 per pound (41% increase) in 2021 (Figure 4; Table S9). 

**Figure 5:**
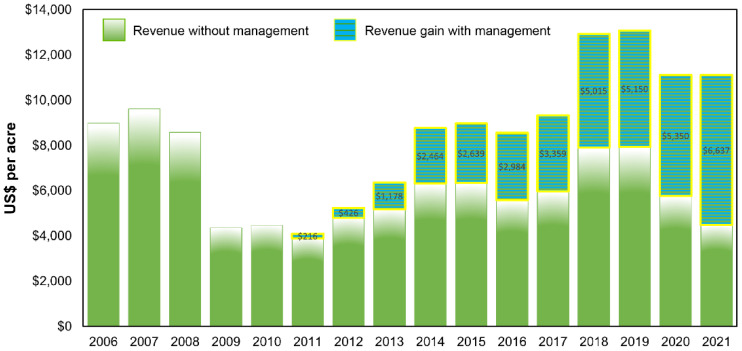
Average revenue (USD per acre) for Hawaii-grown coffee without CBB management from 2006 to 2021 and gain in revenue due to all combined CBB management beginning in 2011.

From 2006 to 2021, coffee revenue ranged from a high of USD 13,078 per acre in 2019 to a low of USD 4096 per acre in 2011 (Figure 5; Table S10). In the five years prior to the CBB invasion the observed average revenue was USD 7196 per acre, compared to an average of USD 6683 per acre in the five years post-invasion (7% decrease). Revenue gains rose from USD 216 per acre (5% increase) in 2011 to USD 6637 per acre (60% increase) in 2021 (Figure 5; Table S10). 

**Figure 6:**
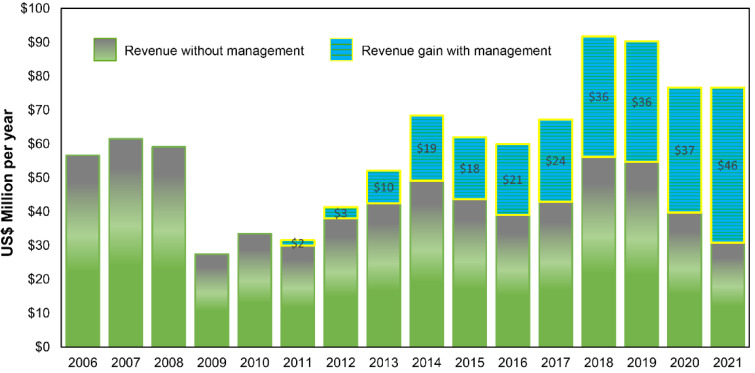
Average statewide revenue (USD million per year) for Hawaii-grown coffee without CBB management from 2006 to 2021 and gain in statewide revenue due to all combined CBB management beginning in 2011.

Lastly, during the 16-year sampling period the observed annual statewide revenue from coffee ranged from a low of USD 27.41 M in 2009 to a high of USD 91.79 M in 2018 (Figure 6; Table S11). Prior to the CBB invasion the observed average statewide revenue was USD 47.63 M annually, compared to an average of USD 51.05 M annually in the five years post-invasion (7% increase). Annual total revenue gains rose from USD 1.66 M per year (5.3% increase) in 2011 to USD 45.79 M per year (59.8% increase) in 2021 (Figure 6; Table S11). 

**Figure 7:**
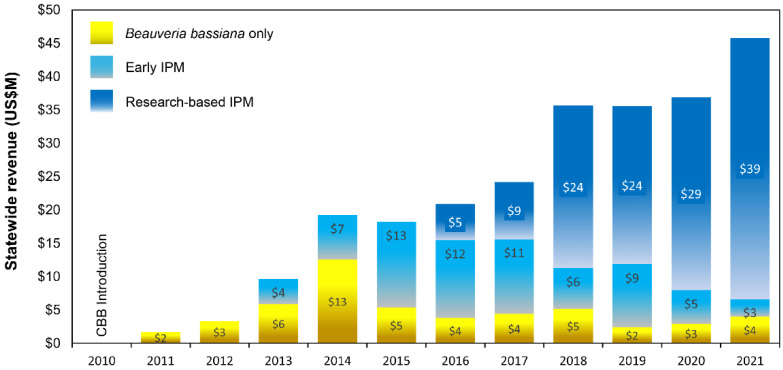
Gain in statewide revenue (USD millions) for Hawaii-grown coffee using three CBB management strategies: *B. bassiana* only (introduced in 2011), early IPM (introduced in 2013), and research-based IPM (introduced in 2016).

Gains to farmers from using *B. bassiana* products only ranged from ~USD 2–13 M between 2011 and 2021, with the highest gains observed in 2013 (Figure 7). Similarly, gains to farmers adopting early IPM recommendations from 2013 to 2021 ranged from ~USD 3–13 M, with the highest gains observed in 2015 (Figure 7). From 2016 to 2021, farmers adopting research-based IPM recommendations enjoyed gains of USD 5–39 M per year, with the highest gains observed in 2021 (Figure 7). During this same period (2016–2021), farmers continuing to follow early IPM recommendations had annual gains of USD 3–12 M, while farmers continuing to use only *B. bassiana* had marginal annual gains of USD 2–5 M (Figure 7; Table S12). 

## 4. Discussion

This study estimated the economic benefits from CBB management in Hawaii over the last 11 years (2011 to 2021) through a collaboration between coffee growers, industry representatives, scientists, extension educators, and economists. We used reported data on coffee acreage in combination with detection dates and expert knowledge to estimate CBB spread across the Hawaiian Islands and to inform model assumptions. Then, adoption rates of management types were based on Roger’s theory of technology adoption. These management types included: (1) the use of the entomopathogenic fungus *B. bassiana* alone, starting in 2011; (2) early IPM that combined monitoring and sanitation with spraying *B. bassiana*, starting in 2013; and (3) the use of research-based IPM that focused on improving monitoring techniques, optimizing sprays of *B. bassiana*, and assessing the impact and feasibility of cultural controls, starting in 2016. Finally, adjustments in yield and prices were based on management effectiveness and bean quality. 

Over a 10-year period, management of CBB-infested acreage in Hawaii has increased from 8% in 2011 to 59% in 2021. During this same period, coffee yields increased by 32%, the price per pound increased by 41%, and revenue per acre increased by 60% due to the improvement of CBB management technology. From 2011 to 2021, the cumulative gain in revenue was USD 251 M with the highest benefit coming from research-based IPM at USD 130 M, followed by early IPM at USD 69 M, and *B. bassiana* at USD 50 M. Annually, when all three management types are adopted, the highest return is from research-based IPM. 

Findings from Hawaii-specific studies on CBB management were first incorporated into the 2016 IPM guidelines, marking the beginning of research-based IPM strategies for CBB in Hawaii. This was followed by the initiation of a five-year area-wide IPM grant in 2017, with the aim of developing, testing, and implementing suites of control measures that were specifically tailored to Hawaii’s unique and highly variable coffee-growing landscape. Under this grant, multiple research projects were initiated to simplify monitoring strategies, gain knowledge of CBB biology under varying environmental conditions, determine the feasibility of cultural controls such as frequent and efficient harvesting and strip-picking in different growing regions, optimize the use of *B. bassiana*, and compare the economics of various control strategies. Collectively, this project produced >25 peer-reviewed journal articles and six cooperative extension publications and demonstration videos. The findings were shared at multiple in-person and virtual conferences, newsletters, as well as an extension website. 

As a result, an improved location-specific research-based IPM was developed and implemented over the years that followed. IPM adoption in Hawaii was facilitated by grower associations and industry to provide long-term funding and extension support. Briefly, the research-based CBB IPM program is defined by four major practices: (1) field sanitation including pruning, weed removal, strip-picking, and stumping by blocks; (2) monitoring CBB populations using traps and/or a simplified method of surveying for berry infestation, (3) applying *B. bassiana* early in the season during peak CBB flight activity, and (4) frequent and efficient harvesting [41]. Our findings demonstrate that both research and outreach are critical for developing and implementing effective IPM strategies. Scientists play a key role in anticipating and identifying pest problems, developing preventative and curative strategies, and driving technological developments by identifying new management tools and performing preliminary investigations. Scientists and extension educators must then work together to change grower perspectives on traditional methods of pest control by demonstrating the value of new strategies and technologies. The implementation of IPM strategies can be facilitated by grower associations and industry representatives to effectively disseminate this information through traditional and modern communication tools and strategies. 

## Figures and Tables

**Table 1:** Observed statewide average coffee yield (parchment pounds per acre) from 2006 to 2021, and statewide yield based on various management scenarios.

Year (t)	Statewide Average Yield (Y_t_)	*Beauveria bassiana* Only (y_Bt_)	Early IPM (y_Ct_)	Research-Based IPM (y_Dt_)	Infested Unmanaged (y_Et_)	Uninfested (y_Ft_)
2006	1175					1175
2007	1172					1172
2008	1261					1261
2009	1208					1208
2010	1173				669	1337
2011	987	960			640	1280
2012	886	869			579	1159
2013	1024	962	1090		641	1282
2014	962	857	971		571	1143
2015	957	840	952		560	1120
2016	771	673	763	853	449	897
2017	840	726	822	919	484	967
2018	965	811	919	1027	541	1082
2019	928	775	878	981	517	1033
2020 ^1^	949	857	971	1085	571	1142
2021 ^1^	949	918	1041	1163	612	1224

^1^ Hawaii data source: Coffee 01/26/2021 (usda.gov (accessed on 1 December 2021)).

**Table 2:** Hawaii statewide average coffee price (USD) per pound for parchment coffee from 2008 to 2021, and statewide price based on various management scenarios.

Year (t)	Statewide Average Price (P_t_)	*B. bassiana* Only (p_Bt_)	Early IPM (p_Ct_)	Research-Based IPM (p_Dt_)	Infested Unmanaged (p_Et_)	Uninfested (p_Ft_)
2008	6.80					6.80
2009	3.60					3.60
2010	3.80				1.85	4.12
2011	4.15	4.30			2.22	4.94
2012	5.90	6.14			3.17	7.05
2013	6.20	6.18	6.62		3.19	7.10
2014	9.12	8.66	9.29		4.48	9.95
2015	9.38	8.85	9.49		4.58	10.17
2016	11.10	10.42	11.17	11.97	5.39	11.97
2017	11.10	10.34	11.09	11.88	5.35	11.88
2018	13.40	12.27	13.15	14.09	6.34	14.09
2019	14.10	12.87	13.79	14.78	6.65	14.78
2020	11.70 ^1^	11.23	12.03	12.90	5.80	12.90
2021	11.70 ^1^	11.80	12.65	13.56	6.10	13.56

^1^ Hawaii data source: Coffee 01/26/2021 usda.gov. (USDA-NASS online reports a different value) (accessed on 1 December 2021).

## Data Availability

Data supporting this article will be deposited in the USDA National Agriculture Library upon acceptance.

## Supplementary Materials

The following supporting information can be downloaded at: https://www.mdpi.com/article/10.3390/insects14040350/s1, Table S1: Regional Hawaii coffee acreage (values reported in blue vs. all other values missing); Table S2: Estimated and calibrated regional Hawaii bearing coffee acreage (a_ht_). Values highlighted in blue represent those for which regional estimates were available (see Table S1 for comparison); Table S3: Hawaii regional CBB infested acres (i_ht_) from 2006 to 2021 (for each region (*h*) the year of CBB introduction (τ_h_) is shown in parentheses); Table S4: Parameter values for the proportion of coffee berry borer infested acres managed (υ_jt_) over time using *Beauveria bassiana* only, early IPM, and research-based IPM; Table S5: Baseline coffee acreage (*a*_jt_) distribution in Hawaii from 2006 to 2021 (total bearing acreage is the sum of all infested and uninfested acres, managed acres is the sum of the three IPM scenarios, and infested acres is the sum of managed and unmanaged acres); Table S6: Proportion of total coffee acreage (ω_Jt_) that is infested (I_t_), uninfested (U_t_), managed (*B. bassiana* only = υ_Bt_, early IPM = υ_Ct_, and research-based IPM = υ_Dt_) and unmanaged (N_t_); Table S7: Coffee prices per pound (USD) under the hypothetical scenario without CBB management; Table S8: Observed coffee yield (parchment pounds per acre), yield without any CBB management, and the gain in yield due to all combined types of CBB management; Table S9: Observed price for coffee (USD per pound of parchment), price without CBB management, and price gain due to CBB management; Table S10: Observed average revenue (USD per acre) for Hawaii-grown coffee, average revenue without CBB management, and gain in average revenue due to CBB management; Table S11: Hawaii statewide observed average revenue (USD million per year), average revenue without CBB management, and gain in revenue due to CBB management. Table S12: Observed average Hawaii statewide coffee revenue (USD million per year), revenue without CBB management, and revenue gain from CBB management by management type.
