# Supplementary material for: Economic Benefits from Managing Coffee Berry Borer (Hypothenemus hampei) in Hawaii
Source: Insects. 2023 Apr 1;14(4):350. doi: 10.3390/insects14040350 (PMC10143774; doi:10.3390/insects14040350)
Supplement: Supplementary file 1 [file insects-14-00350-s001.zip › insects-2304216-supplementary.pdf]

Table S1. Regional Hawaii coffee acreage (values reported in blue vs. all other values missing).

Year	Kona	Kau	Puna	Hamakua	Oahu	Maui	Kauai	Molokai
2006								
2007								
2008								
2009								
2010								
2011								
2012								
2013	3800	500		125	160		2413	
2014						390	2450	
2015	3800	660	70		175	420	3000	123
2016		500	70		155	315	2515	
2017								
2018								
2019								
2020								
2021								

Table S2. Estimated and calibrated regional Hawaii bearing coffee acreage (a_{ht}). Values highlighted in blue represent those for which regional estimates were available (see Table S1 for comparison).

Year	Kona	Kau	Puna	Hamakua	Oahu	Maui	Kauai	Molokai
2006	3679	484	68	121	155	363	2512	119
2007	3679	484	68	121	155	363	2512	119
2008	3679	484	68	121	155	363	2512	119
2009	3679	484	68	121	155	363	2512	119
2010	3679	484	68	121	155	363	2512	119
2011	3777	497	70	124	159	373	2579	122
2012	3875	510	71	127	163	382	2646	125
2013	4118	542	76	135	173	406	2615	133
2014	3847	587	71	127	170	395	2480	125
2015	3131	544	58	103	144	346	2472	101
2016	3499	460	64	115	143	290	2316	113
2017	3493	533	64	115	152	345	2385	113
2018	3445	526	63	113	150	340	2352	111
2019	3348	511	62	110	145	330	2286	108
2020	3348	511	62	110	145	330	2286	108
2021	3348	511	62	110	145	330	2286	108

Table S3. Hawaii regional CBB infested acres (i_{ht}) from 2006-2021. For each region (h) the year of CBB introduction (τ_h) is shown in parentheses.

Year	Kona (2010)	Kau (2011)	Puna (2012)	Hamakua (2013)	Oahu (2014)	Maui (2015)	Kauai (2020)	Molokai (2021)
2006	0	0	0	0	0	0	0	0
2007	0	0	0	0	0	0	0	0
2008	0	0	0	0	0	0	0	0
2009	0	0	0	0	0	0	0	0
2010	1839	0	0	0	0	0	0	0
2011	3588	248	0	0	0	0	0	0
2012	3681	484	36	0	0	0	0	0
2013	3913	515	72	68	0	0	0	0
2014	3654	558	67	120	85	0	0	0
2015	2975	517	55	98	137	173	0	0
2016	3324	437	61	109	136	276	0	0
2017	3318	507	61	109	144	327	0	0
2018	3272	499	60	108	142	323	0	0
2019	3180	485	59	105	138	314	0	0
2020	3180	485	59	105	138	314	1143	0
2021	3180	485	59	105	138	314	2171	54

Table S4. Parameter values for the proportion of coffee berry borer infested acres managed (υ_{jt}) over time using *Beauveria bassiana* only, early IPM, and research-based IPM.

	**Management technology**		
	B. bassiana only	Early IPM	Research based IPM
	**Year technology was introduced**		
Year (t)	2011	2013	2016
	υ_{Bt}	υ_{Ct}	υ_{Dt}
<2010	0	0	0
2010, 2011, 2012	M_t/A_t	0	0
2013	$1-\upsilon_{Ct}$	0.16	0
2014	$1-\upsilon_{Ct}$	0.23	0
2015	$1-\upsilon_{Ct}$	0.50	0
2016	0.19	$1-\upsilon_{Bt}-\upsilon_{Dt}$	0.16
2017	0.20	$1-\upsilon_{Bt}-\upsilon_{Dt}$	0.23
2018	0.18	$1-\upsilon_{Bt}-\upsilon_{Dt}$	0.50
2019, 2020, 2021	0.086	$1-\upsilon_{Bt}-\upsilon_{Dt}$	0.50

Table S5. Baseline coffee acreage (a_{jt}) distribution in Hawaii from 2006-2021. Total bearing acreage is the sum of all infested and uninfested acres, managed acres is the sum of the three IPM scenarios, and infested acres is the sum of managed and unmanaged acres.

		**Sums to total acres (A_t)**		**Sums to all managed acres (M_t)**			**Sums to infested acres (I_t)**	
**Year (t)**	**Total bearing acres (A_t)**	Uninfested (U_t)	Infested (I_t)	*B. bassiana* only (X_{Bt})	Early IPM (X_{Ct})	Research based IPM (X_{Dt})	Managed (M_t)	Unmanaged (N_t)
		a_{Ft}	a_{Et}	a_{Bt}	a_{Ct}	a_{Dt}	$a_{Bt} + a_{Ct} + a_{Dt}$	a_{Et} - ($a_{Bt} + a_{Ct} + a_{Dt}$)
2006	6300	6300	0				0	0
2007	6400	6400	0				0	0
2008	6900	6900	0				0	0
2009	6300	6300	0				0	0
2010	7500	5661	1839				0	1839
2011	7700	3864	3836	614			614	3223
2012	7900	3699	4201	964			964	3237
2013	8200	3633	4567	1510	731		2241	2326
2014	7800	3316	4484	2582	1031		3613	871
2015	6900	2946	3954	1110	1977		3087	867
2016	7000	2657	4343	825	1914	695	3434	909
2017	7200	2733	4467	893	1720	1027	3641	826
2018	7100	2695	4405	793	705	2202	3700	705
2019	6900	2619	4281	368	1087	2140	3596	685
2020	6900[1]	1476	5424	466	600	2712	3779	1645
2021	6900[1]	394	6506	560	283	3253	4095	2411

[1]Data source: Coffee 01/26/2021 usda.gov (USDA-NASS online reports a different value).

Table S6. Proportion of total coffee acreage (ω_{Jt}) that is infested (I_t), uninfested (U_t), managed (*B. bassiana* only = υ_{Bt}, early IPM = υ_{Ct}, and research-based IPM = υ_{Dt}) and unmanaged (N_t).

Management type (j)	ω_{Jt}	Infestation Status	Management Status
**B**	$\upsilon_{Bt}I_t/A_t$	Infested	Managed
**C**	$\upsilon_{Ct}I_t/A_t$	Infested	Managed
**D**	$\upsilon_{Dt}I_t/A_t$	Infested	Managed
**E**	N_t/A_t	Infested	Unmanaged
**F**	U_t/A_t	Uninfested	Management not needed

Table S7. Coffee prices per pound (US$) under the hypothetical scenario without CBB management.

Year (*t*)	Uninfested coffee price (p_{Ft})	Infested unmanaged coffee price (p_{Et})
2006	$7.65	
2007	$8.20	
2008	$6.80	
2009	$3.60	
2010	$4.12	$1.85
2011	$4.94	$2.22
2012	$7.05	$3.17
2013	$7.10	$3.19
2014	$9.95	$4.48
2015	$10.17	$4.58
2016	$11.97	$5.39
2017	$11.88	$5.35
2018	$14.09	$6.34
2019	$14.78	$6.65
2020	$12.90	$5.80
2021	$13.56	$6.10

[1]Index j = Unifested (F) and Infested unmanaged (E); relative yield multiplier (ρ_j) for F = 100%, E = 45%.

Table S8. Observed coffee yield (parchment pounds per acre), yield without any CBB management, and the gain in yield due to all combined types of CBB management.

Year	Observed yield (lbs/acre)	Yield without CBB management (lbs/acre)	Yield gain with CBB management (lbs/acre)	Gain (%)
2006	1175	1175	0	0.0
2007	1172	1172	0	0.0
2008	1261	1261	0	0.0
2009	1208	1208	0	0.0
2010	1173	1173	0	0.0
2011	987	961	26	2.6
2012	886	851	35	4.0
2013	1024	925	99	9.7
2014	962	814	147	15.3
2015	957	799	157	16.5
2016	771	619	152	19.8
2017	840	667	173	20.6
2018	965	746	219	22.7
2019	928	713	215	23.2
2020	949	693	256	27.0
2021	949	647	302	31.8

Table S9. Observed price for coffee (US$ per pound of parchment), price without CBB management, and price gain due to CBB management.

Year	Observed Price (US$/lb)	Price without CBB management (US$/lb)	Price gain with CBB management (US$/lb)	Gain (%)
2006	7.65	7.65	0	0.0
2007	8.20	8.20	0	0.0
2008	6.80	6.80	0	0.0
2009	3.60	3.60	0	0.0
2010	3.80	3.80	0	0.0
2011	4.15	4.04	0.11	2.7
2012	5.90	5.64	0.26	4.3
2013	6.20	5.59	0.61	9.8
2014	9.12	7.74	1.38	15.1
2015	9.38	7.93	1.45	15.5
2016	11.10	9.01	2.09	18.8
2017	11.10	8.94	2.16	19.4
2018	13.40	10.61	2.79	20.8
2019	14.10	11.13	2.97	21.1
2020	11.70	8.30	3.40	29.0
2021	11.70	6.91	4.79	41.0

Table S10. Observed average revenue (US$ per acre) for Hawaii-grown coffee, average revenue without CBB management, and gain in average revenue due to CBB management.

Year	Observed revenue (US$/acre)	Revenue without CBB management (US$/acre)	Revenue gain with CBB management (US$/acre)	Gain (%)
2006	8,986	8,986	0	0
2007	9,609	9,609	0	0
2008	8,574	8,574	0	0
2009	4,350	4,350	0	0
2010	4,459	4,459	0	0
2011	4,096	3,880	216	5
2012	5,228	4,802	426	8
2013	6,351	5,173	1,178	19
2014	8,769	6,305	2,464	28
2015	8,972	6,333	2,639	29
2016	8,563	5,579	2,984	35
2017	9,327	5,968	3,359	36
2018	12,928	7,913	5,015	39
2019	13,078	7,928	5,150	39
2020	11,107	5,756	5,350	48
2021	11,107	4,470	6,637	60

Table S11. Hawaii Statewide observed average revenue (US$ million per year), average revenue without CBB management, and gain in revenue due to CBB management.

Year	Observed revenue (US$M/year)	Revenue without management (US$M/year)	Revenue gain (US$M/year)	Gain (%)
2006	56.61	56.61	0	0.0
2007	61.5	61.50	0	0.0
2008	59.2	59.16	0	0.0
2009	27.4	27.41	0	0.0
2010	33.44	33.44	0	0.0
2011	31.54	29.88	1.66	5.3
2012	41.3	37.93	3.37	8.2
2013	52.1	42.42	9.66	18.5
2014	68.4	49.18	19.22	28.1
2015	61.9	43.70	18.21	29.4
2016	59.9	39.05	20.89	34.8
2017	67.2	42.97	24.19	36.0
2018	91.8	56.18	35.61	38.8
2019	90.2	54.71	35.53	39.4
2020	76.6	39.72	36.92	48.2
2021	76.6	30.84	45.79	59.8

Table S12. Observed average Hawaii Statewide coffee revenue (US$ million per year), revenue without CBB management, and revenue gain from CBB management by management type.

**Year**	**Observed revenue**	**Revenue without CBB management**	**Revenue Gain**			**Gain (%)**
			B. bassiana only	Early IPM	Research-based IPM	
2006	56.61	56.61				0
2007	61.50	61.50				0
2008	59.16	59.16				0
2009	27.41	27.41				0
2010	33.44	33.44				0
2011	31.54	29.88	1.66	0		5
2012	41.30	37.93	3.37	0		8
2013	52.08	42.42	5.88	3.78		19
2014	68.40	49.18	12.56	6.66		28
2015	61.91	43.70	5.41	12.80		29
2016	59.94	39.05	3.79	11.68	5.41	35
2017	67.16	42.97	4.39	11.23	8.56	36
2018	91.79	56.18	5.17	6.10	24.34	39
2019	90.24	54.71	2.41	9.43	23.70	39
2020	76.64	39.72	2.94	5.02	28.96	48
2021	76.64	30.84	3.97	2.66	39.16	60
